# Phenolics-Rich Extracts of Dietary Plants as Regulators of Fructose Uptake in Caco-2 Cells via GLUT5 Involvement

**DOI:** 10.3390/molecules26164745

**Published:** 2021-08-05

**Authors:** Małgorzata Zakłos-Szyda, Nina Pietrzyk, Agnieszka Kowalska-Baron, Adriana Nowak, Katarzyna Chałaśkiewicz, Marcin Ratajewski, Grażyna Budryn, Maria Koziołkiewicz

**Affiliations:** 1 Faculty of Biotechnology and Food Sciences, Institute of Molecular and Industrial Biotechnology, Lodz University of Technology, Stefanowskiego 2/22, 90-537 Łódź, Poland; nina.pietrzyk@dokt.p.lodz.pl (N.P.); katarzyna.chalaskiewicz@dokt.p.lodz.pl (K.C.); maria.koziolkiewicz@p.lodz.pl (M.K.); 2 Faculty of Biotechnology and Food Sciences, Institute of Natural Products and Cosmetics, Lodz University of Technology, Stefanowskiego 2/22, 90-537 Łódź, Poland; agnieszka.kowalska-baron@p.lodz.pl; 3 Department of Environmental Biotechnology, Lodz University of Technology, Wólczańska 171/173, 90-924 Łódź, Poland; adriana.nowak@p.lodz.pl; 4 Institute of Medical Biology, Laboratory of Epigenetics, Polish Academy of Sciences, Tylna 3a, 90-364 Łódź, Poland; mratajewski@cbm.pan.pl; 5 Faculty of Biotechnology and Food Sciences, Institute of Food Technology and Analysis, Lodz University of Technology, Stefanowskiego 2/22, 90-537 Łódź, Poland; grazyna.budryn@p.lodz.pl

**Keywords:** GLUT5, fructose, phenolic compounds, Caco-2

## Abstract

The latest data link the chronic consumption of large amounts of fructose present in food with the generation of hypertension and disturbances in carbohydrate and lipid metabolism, which promote the development of obesity, non-alcoholic fatty liver disease, insulin resistance, and type 2 diabetes. This effect is possible after fructose is absorbed by the small intestine cells and, to a lesser extent, by hepatocytes. Fructose transport is dependent on proteins from the family of glucose transporters (GLUTs), among which GLUT5 selectively absorbs fructose from the intestine. In this study, we examined the effect of four phenolic-rich extracts obtained from *A. graveolens*, *B. juncea*, and *M. chamomilla* on fructose uptake by Caco-2 cells. Extracts from *B. juncea* and *M. chamomilla* most effectively reduced fluorescent fructose analogue (NBDF) accumulation in Caco-2, as well as downregulated GLUT5 protein levels. These preparations were able to decrease the mRNA level of genes encoding transcription factors regulating GLUT5 expression-thioredoxin-interacting protein (TXNIP) and carbohydrate-responsive element-binding protein (ChREBP). Active extracts contained large amounts of apigenin and flavonols. The molecular docking simulation suggested that some of identified phenolic constituents can play an important role in the inhibition of GLUT5-mediated fructose transport.

## 1. Introduction

Fructose is a sugar naturally present in fruits and honey. Fruits and honey were used as occasional sweeteners in the diet of ancestral humans adapted to low levels of fructose [1]. Given that fructose does not stimulate secretion of insulin, many food products today contain significant amounts of fructose and/or glucose–fructose syrup, which increases the level of fructose consumption (the daily dose already exceeds 55–70 g) [1,2]. Since we are not prepared by evolution for large increases in fructose flux, the latest data link the chronic consumption of large amounts of this sugar with the generation of hypertension, and disturbances in carbohydrate and lipid metabolism, which promote the development of obesity, non-alcoholic fatty liver disease, insulin resistance, and type 2 diabetes [3,4,5,6,7]. This effect is possible after fructose absorption by enterocytes in the small intestine and, to a lesser extent, by liver hepatocytes [8]. Fructose transport is dependent on proteins from the family of glucose transporters (GLUTs), among which GLUT2 and GLUT5 mediate intestinal fructose transport from the intestinal lumen into enterocytes and the blood [9]. Transport of fructose at the apical membrane of intestinal cells is primarily mediated by GLUT5, but it can be also mediated by a low-affinity transporter GLUT2, which, additionally to fructose, recognizes glucose and galactose [10]. Fructose taken up by enterocytes is transported across the basolateral membrane into the portal blood by GLUT2, from which is taken up by GLUT2 expressed by hepatocytes. However, recent studies indicate that glucose transporter 8 (GLUT8/SLC2A8) can also mediate fructose transport into hepatocytes and hepatic fructose metabolism [11]. Liver almost exclusively metabolizes up-taken fructose, but this sugar can also be metabolized in the intestinal cells, however to a lesser extent.

The main catabolic pathway of fructose involves the enzyme ketohexokinase (KHK), also known as fructokinase [12]. Fructose ingestion upregulates the level of GLUT5 in enterocytes, whereas deletion of KHK diminishes GLUT5 expression in response to high fructose [13]. The KHK enzyme utilizes ATP to phosphorylate fructose to fructose-1-phosphate, which is further cleaved to glyceraldehyde and dihydroxyacetone phosphate by aldolase B. These triose metabolites become substrates for gluconeogenesis or cellular respiration; however, they largely increase lipogenesis and triglycerides cellular production, leading to the development of steatosis [14]. Furthermore, rapid depletion of AMP resulting from fructose utilization may lead to increased uric acid production and hyperuricemia [12,13,15].

Although GLUT5 is fructose-specific and is not able to transport other carbohydrates [16], still little is known about its function, particularly on physiological substrates and inhibitors [17]. GLUT5 belongs to the major facilitator superfamily, members of which are composed of approximately 500 residues and share common features such as 12 putative transmembrane helices and intracellular C and N termini. Its structure is characterized by two domains, containing six helices each, connected by intracellular (ICH) domain loop [18,19]. Based on the crystal structure of rat and bovine GLUT5, it was demonstrated that fructose transport is controlled by rocker switch-type movement and a gated pore mechanism in which the seventh and tenth transmembrane domains are involved [8]. The model of human GLUT5 in inward-facing conformation, obtained by homology modelling in the study of Nomura et al. [8], allowed to indicate residues lining the central fructose-binding site. These residues included Ile169, Gln166, Gln287, Gln288, Asn324, Trp419, Tyr31, His386, Ala395, His418, and Ser391 [8]. A study performed by Ebert K. et al. [20] indicated Gln167, Ile170, Ile174, and also Val293 residues as critical for fructose substrate specificity of GLUT5, which are potentially involved in fructose binding in the fifth, seventh, eighth, tenth, and eleventh transmembrane domain and in the first extracellular and last intracellular loop. Regardless of these results, *N*-[4-(methylsulfonyl)-2-nitrophenyl] -1,3-benzodioxol-5-amine (MSNBA) was identified as a selective and potent inhibitor of fructose transport facilitated by GLUT5 [16]. The results of ligand docking, mutagenesis, and functional studies showed that the MSNBA binding site is near to the active center of the transporter, and that H387 is involved in inhibitor discrimination. The other residues involved in MSNBA binding included Thr171, Ser143, Tyr297, Gln288, Gln289, and Asn294.

Recently, the search for fructose absorption inhibitors capable of direct interaction with GLUTs has started. So far, such activity has been demonstrated for chemically pure flavonoids, such as catechin and epicatechin gallates, kaempferol, quercetin, or apigenin, and also for two multicomponent preparations from chamomile and green tea rich in these phenolics [21,22,23,24,25,26]. Based on this data, we have selected other dietary plants that are a rich source of mentioned polyphenols and also contain a low amount of free simple sugars. Therefore, in the presented study, we evaluated the effect on fructose uptake of three phenolics-rich extracts obtained from celery root (*Apium graveolens* L., var. rapaceum, Talar, AGE), and mustard leaves of *Brassica juncea* var. Green giant (BJE1) and leaves of *Brassica juncea* var. Red giant (BJE2). These plants are rich source in different bioactive phytochemicals and possess medicinal herb properties protecting against tumors and oxidative damage, and can be quite often found in a human diet as vegetables generating meal’ flavor [27,28,29,30,31,32], but their potential in regulation of fructose metabolism has not been examined, yet. As a positive control for our studies, the extract obtained from chamomile dried flowers (*Matricaria chamomilla*, MCE) was used. *M. chamomilla* is traditionally used as a tea for digestive improvement and protection against inflammation, but more importantly, it is one of the few plants with a proved ability to attenuate fructose transport via inhibition of GLUT5 and GLUT2 [22,33]. As a cellular model in this study, human originated Caco-2 cells were used. These cells resemble enterocytes and are widely employed in studies exploring nutrients biological activity or bioavailability, as well as sugars absorption [34,35]. We determined the effect of phenolic extracts on fluorescent fructose analogue uptake-1-deoxy-1-[(7-nitro-2,1,3-benzoxadiazol-4-yl)amino]-d-fructose (NBDF) [36]. The studies were also focused on the extracts influence on GLUT5 protein levels and possible molecular basis of the GLUT5-ligand interactions. In order to improve our understanding of substrate and inhibitor recognition by GLUT5, we have performed molecular docking simulations of fluorescent fructose analogue used in the fructose uptake experiments (NBDF), as well as the selected phenolic compounds present in extracts, to bovine GLUT5 which shares ~81% sequence identity to human GLUT5 [8]. To the best of our knowledge, this is the first study demonstrating the cell-based in vitro activity of phenolics-rich extracts from *Apium graveolens*, *Brassica juncea*, and *Matricaria chamomilla* on fructose uptake via GLUT5, as well as their regulation of GLUT5 expression with the involvement of carbohydrate-responsive element-binding protein (ChREBP) and thioredoxin-interacting protein (TXNIP). The obtained outcomes contribute to a better understanding of GLUT5 function and its activity regulation by phytocompounds present in the human diet. These results provide new knowledge about the possibility of using *Brassica juncea* and *Matricaria chamomilla* as food components that minimize the harmful effects of fructose via inhibition of its uptake. Still, future studies should be focused on the phenolic-rich extracts activity after their in vitro digestion process.

## 2. Results

### 2.1. The Effect of Extracts on Cell Metabolic Activity

First, the impact of preparations on metabolic activity of Caco-2 cells was studied. A biological material was used as ethanol extracts obtained from the plants, as indicated above, as well as the purified fractions rich in phenolics obtained from the ethanol extracts with SPE method (p). To determine cytotoxic potential of these preparations, cells were incubated for 24 h in the presence of these preparations at the range of 0–10 mg/mL (mg of freeze-dried extract/mL).

**Figure 1:**
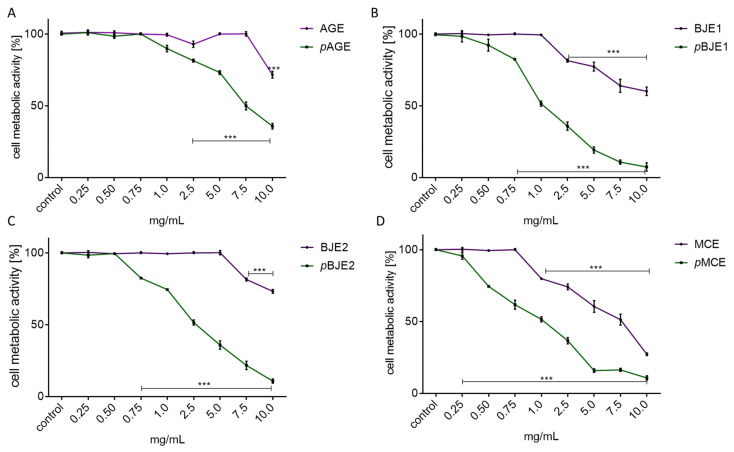
The influence of extracts (*Apium graveolens*, AGE) (**A**), leaf mustard 1 (*Brassica juncea* 1, BJE1) (**B**), leaf mustard 2 (*Brassica juncea* 2, BJE2) (**C**) and chamomile (*Matricaria chamomilla*, MCE) (**D**) on Caco-2 cells’ metabolic activity after their 24-h incubation with ethanolic extracts and the SPE-purified extracts (p) at 0.25–10 mg/mL concentration; control cells were not exposed to any compound except vehicle; values are means ± standard deviations from at least three independent experiments, *n* ≥ 9; statistical significance was calculated versus control cells (untreated) with *** *p* ≤ 0.001.

As it is presented in Figure 1, ethanolic extracts were less cytotoxic than phenolic-rich fractions (p). The highest cytotoxicity was revealed for the MCE extract with IC_50_ = 7.5 mg/mL. The IC_50_ value for AGE and BJE1/2 extracts could not be achieved within the range of the concentrations used in the experiment (10 mg/mL). In the case of extracts purified with solid-phase extraction (p), the highest cytotoxicity was also observed for pMCE, where 0.5 mg/mL decreased cellular activity by almost 25% compared to the control cells. Comparison of the IC_50_ concentration values obtained for the preparations studied allows to determine their impact on metabolic activity, which is pMCE > pBJE1 > pBJE2 > pAGE. The highest non-cytotoxic concentration (IC_0_) values determined for each preparation are summarized in Table 1.

In accordance with previous data [37], the purified extracts were more active than their ethanolic counterparts, and the IC_0_ parameters are as follows: 0.75 mg/mL for pAGE, 0.5 mg/mL for pBJE2, 0.25 mg/mL for pBJE1, and 0.25 mg/mL for pMCE. To directly compare the biological effects of the extracts, a 0.25 mg/mL concentration of each preparation was chosen for further studies.

### 2.2. The Effect of Extracts on Intracellular Reactive Oxygen Species Production and DNA Repair

**Figure 2:**
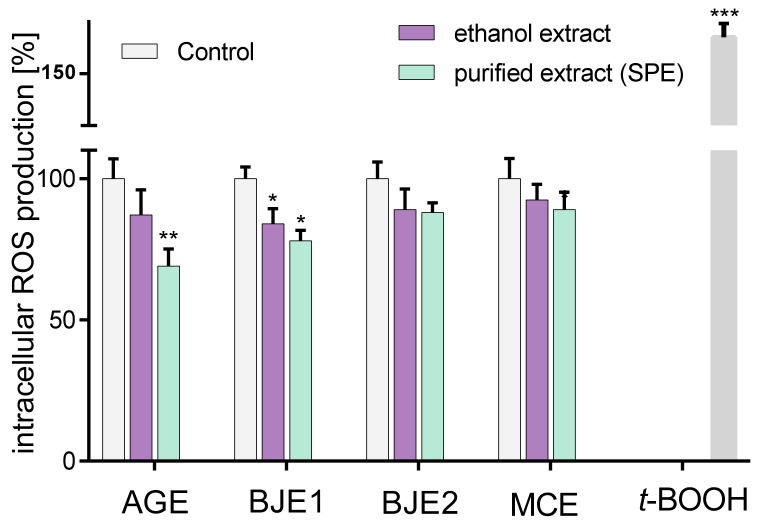
The influence of the ethanolic extracts and the SPE-purified extracts (p) of *Apium graveolens* (AGE) (A), *Brassica juncea* 1 (BJE1) (B), *Brassica juncea* 2 (BJE2) (C) and *Matricaria chamomilla* (MCE) (D) at 0.25 mg/mL concentration on intracellular ROS generation in Caco-2 cells after 24-h incubation, quantified with DCFH-DA assay; control cells were not exposed to any compound except vehicle; values are means ± standard deviations from at least three independent experiments, *n* ≥ 9; statistical significance was calculated versus control cells (untreated) with * *p* ≤ 0.05, ** *p* ≤ 0.01, *** *p* ≤ 0.001; 500 µM t-BOOH was used as positive control.

Reactive oxygen species (ROS) are important signaling molecules in cells; however, their excessive formation leads to oxidative stress causing the damage and death of cells. Since epithelial cells of gastrointestinal tract are constantly exposed to luminal oxidants from ingested foods, in the next step the antioxidant potential of the extracts was determined. The results collected in Figure 2 show that all ethanolic extracts at a non-cytotoxic concentration (0.25 mg/mL) declined intracellular ROS level by 10–15% in comparison to the control cells, and the most efficient result was observed for BJE1. However, the purified extracts were more efficient as oxidative stress reducers than the ethanolic extracts. In this case, all preparations (containing purified phenolic compounds) decreased intracellular ROS level by 15–30%, where the most significant reduction was observed for pAGE. Observed reduction in intracellular ROS is related to antioxidant ability, which can protect cells against damage via conversion of free radicals into non-radical compounds, breaking the chain reaction of protein or lipid oxidation.

**Figure 3:**
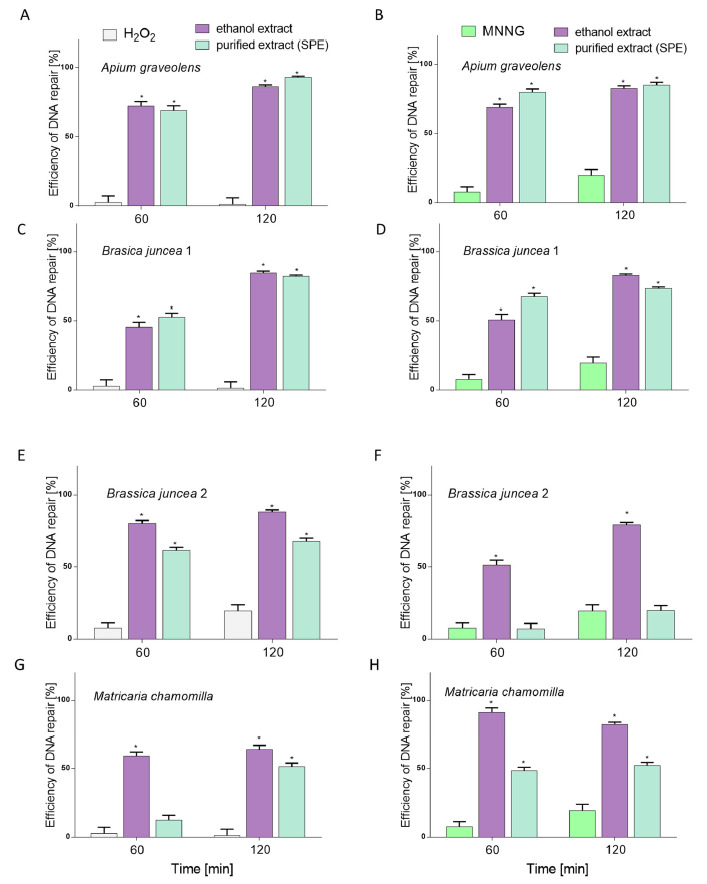
Efficiency of DNA repair (%) in Caco-2 cells exposed to 25 µM H_2_O_2_ (**A**,**C**,**E**,**G**) or to 6.8 µM MNNG (**B**,**D**,**F**,**H**) for 10 min. Then cells were post-incubated for 60 and 120 min with the ethanolic extracts and the SPE-purified extracts (p) of *Apium graveolens* (AGE) (**A**,**B**), *Brassica juncea* 1 (BJE1) (**C**,**D**), *Brassica juncea* 2 (BJE2) (**E**,**F**) and *Matricaria chamomilla* (MCE) (**G**,**H**) at 0.25 mg/mL concentration. The number of cells analyzed for each time-interval was 50. Error bars denote S.E.M. ANOVA (*p* < 0.05). * Significantly different from the positive control.

Next, the cytoprotective effect of the preparations against cellular DNA damage was evaluated after Caco-2 cells challenged with hydroperoxide (H_2_O_2_) or methylnitronitrosoguanidine (MNNG). In the case of positive controls (cells not treated with plant extracts), after 120 min incubation in comparison to the initial point, approximately 20% efficiency of DNA repair was observed for MNNG, while for H_2_O_2_ it was approximately 2% (Figure 3). All extracts induced DNA repair in a statistically significant manner (*p* < 0.05), except for purified extract from BJE2 (after induction of DNA damage with MNNG), where the lowest (c.a. 10 and 20% after 60 and 120 min, respectively) efficacy of DNA repair was demonstrated. Remaining extracts very efficiently induced DNA repairs, and AGE preparations appeared to be the strongest DNA repair inducers, already after 60 min of incubation, in case of both mutagens. The induction was from approximately 70–80% after 60 min to 80–95% after 120 min (*p* < 0.05).

**Figure 4:**
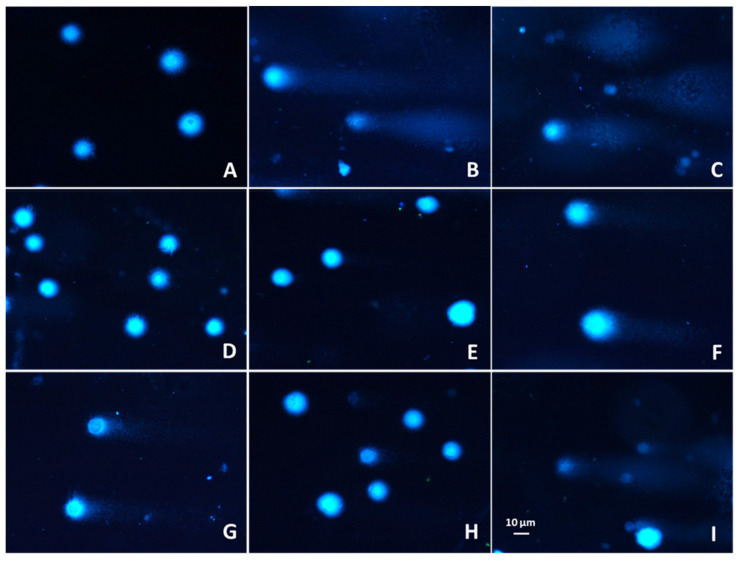
Representative photos of Caco-2 comets after staining with 1 mg/mL DAPI, where the head is composed of intact DNA and the tail consists of damaged DNA (single-strand or double-strand breaks): (**A**)-negative control (cells treated with the vehicle); (**B**)-cells treated with H_2_O_2_ (25 µM); (**C**)-cells treated with MNNG (6.8 µM). Comets visualized after 120 min cells post-treatment with H_2_O_2_ and AGE (**D**), pAGE (**E**), BJE2 (**F**), pBJE2 (**G**). Comets visualized after 120 min cells post-treatment with MNNG and BJE1 (**H**), pBJE1 (**I**). *Apium graveolens* extract, AGE; *Brassica juncea* extract, BJE, purified extracts (p). Fluorescence microscopy (Nikon, Tokyo, Japan); objective 20×.

In the case of BJE1, after induction of DNA damage by H_2_O_2_, the greatest effectiveness of DNA repair (approximately 80% after 60 min and 90% after 120 min) was observed for the ethanol extract. Example images of typical comets are presented in Figure 4. Surprisingly, two of the studied purified extracts, pBJE2 and pMCE, had a lower effect on the DNA repairment in cells challenged with both DNA-damaging agents than the corresponding ethanolic extracts. Still, all the preparations revealed cytoprotective potential against chemically induced DNA damage.

### 2.3. The Influence of Extracts on Fructose Uptake

**Figure 5:**
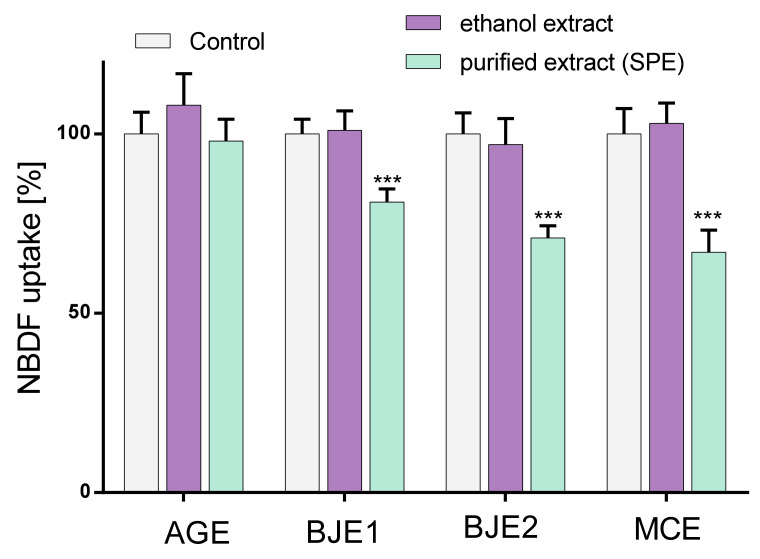
The influence of the extracts from *Apium graveolens* (AGE), *Matricaria chamomilla* (MCE), *Brassica juncea* 1/2 (BJE1/2) and the purified extracts (p) at 0.25 mg/mL concentration on NBD-fructose uptake by Caco-2 cells after 24-h exposure; cells were incubated with 100 µM NBDF before fluorescence measurement; control cells were not exposed to any compound except vehicle; values are means ± standard deviations from at least three independent experiments, *n* ≥ 9; statistical significance was calculated versus control cells (untreated) with *** *p* ≤ 0.001.

To check the influence of extracts on fructose uptake by Caco-2 cells, the fluorescent fructose analogue was used-1-(7-nitro-1,2,3-benzadiazole)-fructose (NBDF) [36]. Among the preparations studied, ethanol extracts had no influence on NBDF uptake by Caco-2 cells (Figure 5). AGE and pAGE had no effect on NBD-fructose level at the concentration studied. Both samples obtained from *Brassica juncea*, pBJE1 and pBJE2, inhibited cellular uptake of NBDF by almost 20 and 30%, respectively. The most effective inhibition was observed for the purified extract from *Matricaria chamomilla*, pMCE, which reduced NBDF fluorescence level by almost 30%.

**Figure 6:**
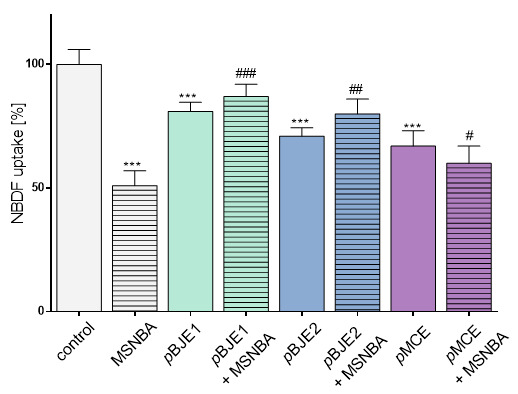
The effect of the purified extracts of *Brassica juncea* 1/2 (pBJE1/2) and *Matricaria chamomilla* (pMCE) used at 0.25 mg/mL concentration on NBD-fructose (100 µM) uptake by Caco-2 cells after 24-h exposure; as GLUT5 inhibitor 50 µM MSNBA was used (as a single compound or simultaneously with the preparations); control cells were not exposed to any compound except vehicle; values are means ± standard deviations from at least three independent experiments, *n* ≥ 9; statistical significance was calculated versus control cells (untreated) with *** *p* ≤ 0.001; statistical significance calculated versus cells treated with MSNBA with ^#^ *p* ≤ 0.05, ^##^ *p* ≤ 0.01, ^###^ *p* ≤ 0.001.

The selective fructose transport across cell membrane is carried out by GLUT5 transporter and this transporter is also involved in cellular NBDF uptake [36]. Therefore, to better characterize the effect of pBJE1/2 and pMCE constituents on GLUT5 mediated transport, the next experiment was assessed in the presence of the selective GLUT5 inhibitor-*N*-[4-(methylsulfonyl)-2-nitrophenyl]-1,3-benzodioxol-5-amine (MSNBA) [16]. As it is shown in Figure 6, preliminary incubation of the cells with 50 µM MSNBA decreased NBDF uptake to about 50% of the control. When used in the mixture with MSNBA, the pBJE1/2 preparations caused a less marked decrease in the uptake of NBDF than MSNBA alone. This result suggests that the tested preparations inhibit GLUT5-mediated uptake of NBDF. On the other hand, pMCE and MSNBA slightly deepened reduction in NBDF uptake, which may suggest their influence on GLUT5 protein, as well as on transporter other than GLUT5, i.e., GLUT2, which is able to recognize fructose and glucose [35].

### 2.4. The Effects of Extracts on GLUT5 Level

Since the reduction in NBDF uptake by Caco-2 cells may result not only from direct inhibition of the GLUT5 activity but can also be caused via activation of other mechanisms, the impact of extracts on the GLUT5 protein level was determined.

**Figure 7:**
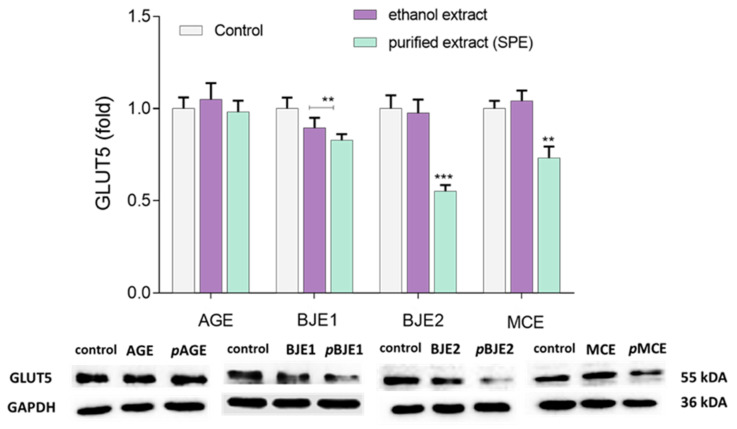
Effect of the ethanolic extracts and the extracts purified with SPE (p) of *Apium graveolens* (AGE), *Matricaria chamomilla* (MCE), and *Brassica juncea* 1/2 (BJE1/2) (0.25 mg/mL) on the relative level of GLUT5 protein in Caco-2 cells after 36-h incubation determined by Western blot analysis control cells were not exposed to any compound (but vehicle); values are means ± standard deviations, *n* ≥ 4; statistical significance was calculated between treatment and control cells (untreated), ** *p* ≤ 0.01, *** *p* ≤ 0.001.

Western blot analysis showed (Figure 7) that the extracts from *Apium graveolens* had no effect on the GLUT5 protein level. Among other ethanol extracts, only the BJE1 decreased the GLUT protein expression by 10%. On the other hand, the preparations obtained via extraction into the solid phase (except pAGE) significantly decreased the expression of GLUT5. pBJE2 downregulated GLUT5 by almost 45%, whereas pBJE1 by 20% of the control. The purified extract from *Matricaria chamomilla* decreased the GLUT5 expression by 25%.

**Figure 8:**
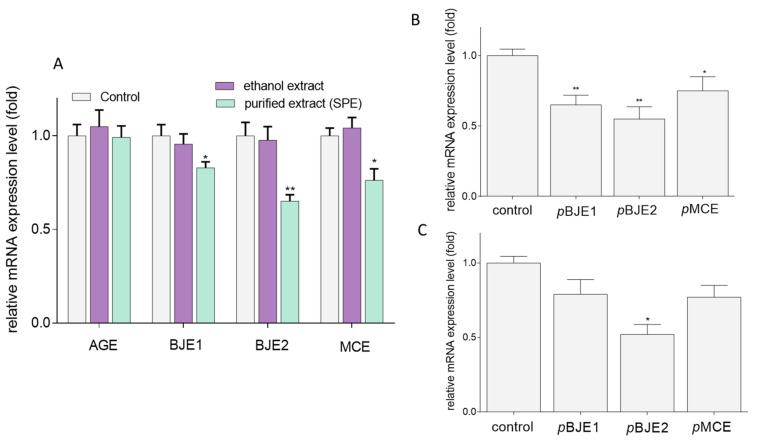
Effect of the ethanolic extracts and the extracts purified with SPE (p) of *Apium graveolens* (AGE), *Brassica juncea* 1/2 (BJE1/2), and *Matricaria chamomilla* (MCE) (0.25 mg/mL) on the mRNA level of GLUT5 (**A**) TXNIP, (**B**) ChREBP, and (**C**) in Caco-2 cells after 24-h incubation determined by real-time PCR and normalized using GAPDH as a reference gene; control cells were not exposed to any compound but vehicle; values are means ± standard deviations, *n* ≥ 4; statistical significance was calculated versus control cells (untreated), * *p* ≤ 0.05, ** *p* ≤ 0.01.

These results were additionally confirmed by analysis of mRNA encoded by GLUT5 gene. In regard to previous studies, the purified extracts rich in phenolic compounds presented a higher impact on Caco-2 cells than the ethanol crude extracts. As it is demonstrated in Figure 8A, the *GLUT5* gene expression level was reduced to 20, 40, and 25% of the control when the cells were treated with pBJE1, pBJE2 and pMCE, respectively. Since various factors can be involved in fructose uptake via GLUT5, the mRNA levels of other selected genes encoding thioredoxin-interacting protein (TXNIP) and carbohydrate-responsive element-binding protein (ChREBP) were determined. Following Caco-2 cells treatment with the purified pBJE1/2 extracts (Figure 8B), the TXNIP mRNA level decreased to 65–70%, while pMCE declined its level by 25%. As shown in Figure 8C, the pBJE2 preparation suppressed the expression of *ChREBP* to 55% of the control. Although not statistically significant, this tendency was also observed for pBJE1 and pMCE.

### 2.5. Phenolic Compounds Profile and Content Determined by UPLC Method

In order to relate the observed activity with the presence of specific phenolic compounds, an analysis of the extracts after purification with solid-phase extraction (SPE) was performed. Phenolic composition of pAGE, pBJE1/2, and pMCE was determined by UPLC method, and the results are shown in Table 2. Based on a comparison of retention times and UV-vis spectra (200–600 nm) with the data for standards in the tested extracts, 23 phenolic compounds were marked with the significant differences (*p* ≤ 0.05) in the content of individual phenolic constituents. The identified phenolic compounds belong to the groups of hydroxybenzoic acids (HBA), hydroxycinnamic acids (HCA), flavanols, flavonols, and flavons. The examined extracts have shown significant qualitative and quantitative diversity. Among the purified extracts, the pAGE contained the highest amount of total phenolic compounds with the value 680.20 mg/g of extract, whereas pMCE was the poorest source of phenolics (153.07 mg/g of extract). In pAGE, flavanols were the dominant group of phenolic compounds (42% sum of total phenolics) with procyanidin C1 (37%) as the main representative. Among flavones, apigenin-7-*O*-glucoside was identified as the main phenolic component (44%). Additionally, among flavanols, procyanidin B2, (-)-epicatechin, epigallocatechin-3-gallate (EGCG), and epicatechin-3-gallate (ECG) were identified. In comparison to other tested extracts, flavonols were not identified in pAGE. In the pMCE and pBJE1/2 preparations, glucosides of quercetin, kaempferol and isorhamnetin, isorhamnetin-3-*O*-rutinoside, quercetin-3-*O*-glucuronide, and kaempferol were identified. In the case of pMCE and pBJE1/2, one of the most abundant compounds was apigenin with the concentrations of 40.47 mg/g, 165.81 mg/g, and 199.12 mg/g of extract, respectively. In pMCE, another flavone-luteolin (6.45 mg/g) was also identified. All the tested extracts were also rich in HBA and HCA derivatives, such as gallic acid, 3-coumaric acid, 3,5-dicaffeoylquinic acid, ferulic acid, salicylic acid, and chlorogenic acid (selectively present in pMCE) and sinapic acid (in pBJE2). Ferulic acid was identified in all of the tested extracts, whereas in pMCE, hydroxycinnamic acid was dominant and constituted more than 55% of HCA and HBA derivatives, next to salicylic acid (42%). It is worth noting that even both the *Brassica juncea* extracts differed in quantitative and qualitative content of the identified phenolic compounds. In pBJE1, only gallic and ferulic acids were identified within the HBA and HCA group, whereas apigenin-7-*O*-glucoside and EGCG were not detected. Both the pBJE1 and pBJE2 preparations were more similar in regard to flavonol composition, but pBJE2 had a higher content of rutin (25.28 mg/g), quercetin-3-*O*-glucoside (27.70 mg/g) and kaempferol-3-*O*-glucoside (18.56 mg/g), while pBJE1 was more rich in isorhamnetin-3-*O*-glucoside (9.83 mg/g), isorhamnetin-3-*O*-rutinoside (4.72 mg/g), and kaempferol (30.97 mg/g).

### 2.6. Determination of Phenolic Constituents’ Binding Site in GLUT5 Model

In regard to the results presented above, the phenolics chemical composition determines cytotoxic or cytoprotective potential of studied extracts, as well as the uptake of fructose fluorescent analogue. The richest source of phenolics, pAGE, had no inhibitory effect on NBDF uptake or on GLUT5 mRNA and protein expression levels.

More detailed studies performed in the presence of selective GLUT5 inhibitor revealed that pBJE1/2 and pMCE could potentially contain phenolic constituent/constituents with inhibitory potential against the GLUT5 transporter. In order to improve the understanding of substrate and inhibitor recognition by GLUT5, in the next step the molecular docking simulations of NBDF, MSNBA, as well as selected phenolic compounds identified in the extracts to GLUT5, were performed.

**Figure 9:**
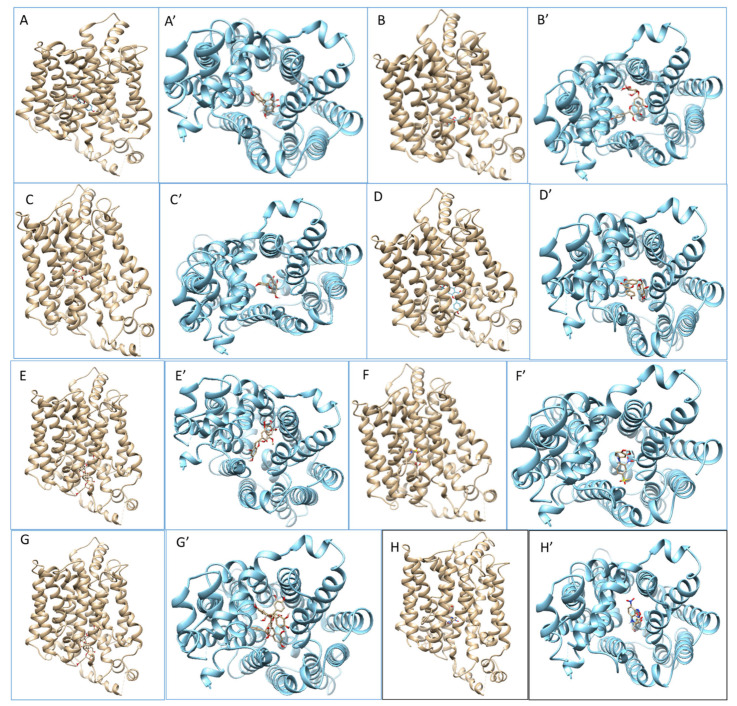
Graphical representation of the lowest energy conformations of GLUT5-ligand complexes obtained from molecular docking simulations: GLUT5-kaempferol (**A**); GLUT5-kaempferol-3-*O*-glycoside (**B**); GLUT5-apigenin (**C**); GLUT5-apigenin-7-*O*-glucoside (**D**); GLUT5-epigallocatechin-3-gallate (ECGC) (**E**); GLUT5-MSNBA (**F**); GLUT5-procyanidin C1 (**G**); GLUT5-NBDF (**H**); vertical view of the GLUT5-ligand complex from the outside is presented with the brown structures of the transporter (picture marked with letter without ’) whereas the corresponding plan view is presented with blue structures of the transporter (picture marked with letter with ’).

**Figure 10:**
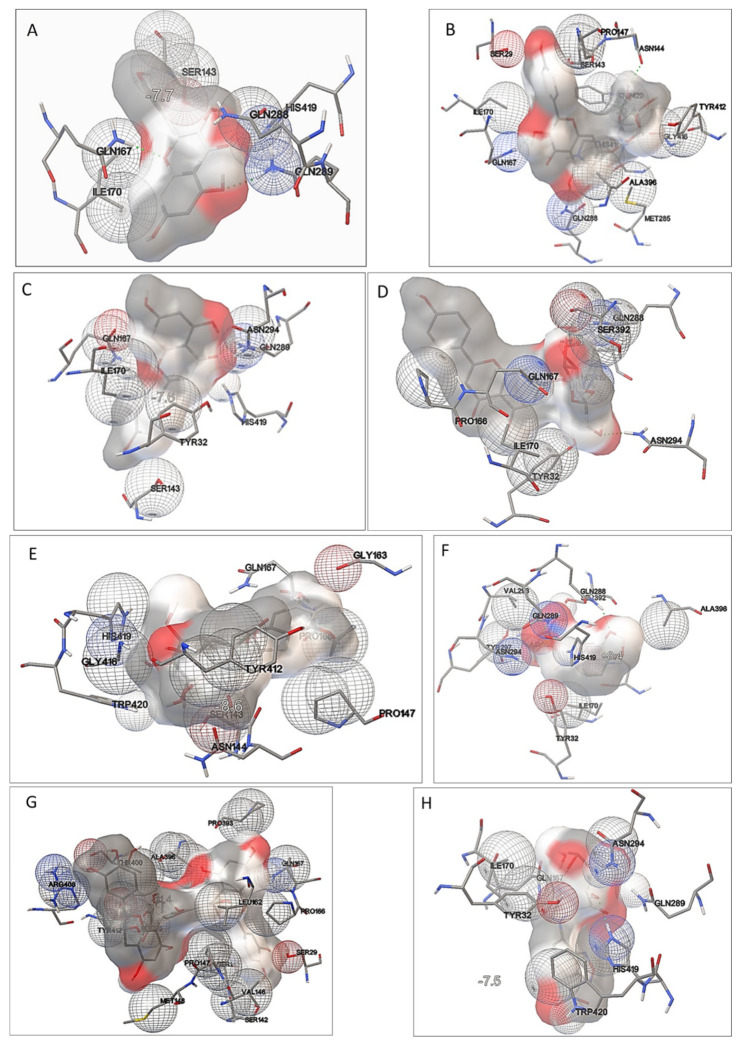
Graphical representation of the binding site of the lowest energy GLUT5-ligand complexes: GLUT5-kaempferol (**A**); GLUT5-kaempferol-3-*O*-glycoside (**B**); GLUT5-apigenin (**C**); GLUT5-apigenin-7-*O*-glucoside (**D**); GLUT5-epigallocatechin-3-gallate (ECGC) (**E**); GLUT5-MSNBA (**F**); GLUT5-procyanidin C1 (**G**); GLUT5-NBDF (**H**).

Graphical representations of the GLUT5-ligand complexes are presented in Figure 9. The compounds tested bound close to the GLUT5 active side, and the residues which were involved in protein-ligand interactions are gathered in Table 3 and presented in Figure 10. The negative values of binding affinities (Table 3) suggest the existence of intermolecular interactions leading to the spontaneous formation of a stable intermolecular complex. The ligands showed similar binding affinities of about-10 kcal/mol. The lowest value of binding energy indicating strong intermolecular interactions was obtained for GLUT5-procyanidin C1 complex, whereas the highest value was determined for the fluorescent fructose analogue NBDF.

Inspection of the amino acid residues in the GLUT5 model interacting with both NBDF and the tested potential inhibitors (Table 3, Figure 10) suggests that all the ligands bind close to the fructose binding site, since most of the interacting residues are those lining the central fructose binding site of GLUT5 (Ile170, Ile174, Gln167, Gln288, Gln289, Asn325, Trp419, Tyr32, His387, Ser392 [8]. Interestingly, NBDF and all the tested potential inhibitors interact with Gln167 and most of them (with the exception of procyanidin C1 and ECGC) also with Ile170 (Table 3). These residues, according to the previous study [8,20], are considered as the key residues involved in fructose transport.

According to [16], the amino acid residues interacting with the highly specific inhibitor of GLUT5-MSNBA include Ser143, Thr171, Tyr297, Asn294, His387, Gln288, and Gln289. Some of these residues (Asn294, Gln288, Gln289, and Tyr297) are also involved in intermolecular interactions in the most stable configuration of GLUT5-MSNBA complex. Moreover, in the case of GLUT5-MSNBA complex, Val293 was also found amongst the interacting residues. The Val293 residue together with Gln167 were identified as the most critical residues that define GLUT5 substrate specificity for fructose [20].

Hydrogen bonding interactions, which increase the stability of the interacting systems, were identified in the complexes of GLUT5 with almost all the tested ligands; only in the case of interaction with ECGC and procyanidin C1 was no hydrogen bond formed. The hydrogen bonding interactions involved: Gln288, Gln167 residues, and kaempferol/kaempferol-3-*O*-glucoside; Gln289 and apigenin; Gln288, Asn294, apigenin-7-*O*-glucoside, Asn325, Gln288, Gln289, and MSNBA; and His419 and NBDF. It should be emphasized that the key residue for fructose transport, Gln167, is involved in hydrogen bonding interactions with kaempferol and kaempferol-3-*O*-glucoside (Table 3). This hydrogen bonding interaction makes Gln67 no longer available for interactions with fructose, which eventually may result in the inhibition of GLUT5-mediated fructose transport.

Our results showed that the extracts rich in kaempferol and kaempferol-3-*O*-glucoside exhibited high inhibitory effects on fructose uptake. The obtained theoretical results demonstrated that kaempferol and kaempferol-3-*O*-glucoside are directly involved in the hydrogen bonding interaction with Gln167, which may result in lesser accessibility of this residue to interact with fructose substrate and, in turn, an inhibition of fructose transport. In the most stable conformations of GLUT5-apigenin and GLUT5- apigenin-7-*O*-glucoside complexes, the hydrogen bonding interactions incorporating other Gln residue (Gln289 and Glu288; Table 3) were present.

Three hydrogen bonding interactions between MSNBA, i.e., Gln288, Gln289, and Asn325, were found in the lowest energy conformation of the GLUT5-MSNBA complex, which is consistent with the previous studies by Thompson et al. [16], showing high inhibitory potential of this compound to fructose transport.

Our experimental findings indicated that the extracts in which procyanidin C1 was present did not exhibit inhibitory effect on fructose uptake. Inspection of binding site of GLUT5-procyanidin C1 complex revealed no Ile170 residue, which was previously indicated as one of the determinants of GLUT 5 specificity to fructose [20]. Moreover, no hydrogen bonds were found in the interacting GLUT5-procyanidin C1 system. Giving a closer look to the geometry of the GLUT5-procyanidin C1 complex (Figure 9G), one can speculate that, due to larger (as compared to the other tested ligands) molecule size of procyanidin C1, this ligand may act as a steric hindrance and hamper the interaction of other inhibitors of higher inhibitory potential with the key residues located in fructose binding site. This may at least partly explain the low inhibitory effect of *Apium graveolens* extracts rich in procyanidin C1 on fructose uptake.

## 3. Discussion

Fructose contribution to the development of metabolic diseases has been studied efficiently [2], therefore there is an increasing need to determine an influence of dietary-plants-derived components on fructose uptake [4]. Among the phytocompounds widely distributed in foods are phenolic compounds, present in large amounts in fruits, vegetables, and beverages. Health-promoting effects of polyphenols are matched with their antioxidant, antimicrobial, anti-inflammatory, and anticancer properties [38], but there is also growing evidence in regard to their influence on intestinal sugar transporters via modulation of their function and alteration of glucose and fructose absorption [24,39]. In this study, we examined the effect of four phenolic-rich extracts obtained from celery root (*Apium graveolens* L., var. rapaceum, Talar, AGE), mustard leaves of *Brassica juncea* var. Green giant (BJE1), and of *Brassica juncea* var. Red giant (BJE2) on fructose uptake by Caco-2 cells. As a positive control, we used extract from chamomile dried flowers (*Matricaria chamomilla*, MCE), which is known fructose uptake inhibitor [22,33].

GLUT5 and GLUT2 are the primary transporters responsible for facilitative absorption of fructose [8] and are strongly expressed in Caco-2 cells [40]. Since GLUT5 selectively uptakes fructose within the apical localization of intestinal cells, we focused on determining the effect of the extracts on this particular transporter. The GLUT5 protein level was determined using Western blot analysis after Caco-2 cells treatment with the extracts at non-cytotoxic concentration. Among them, only the purified extracts from *B. juncea* (pBJE1/2) and *M. chamomilla* (pMCE) decreased the expression of the GLUT5 protein. Ethanol extracts had no influence in this regard, with the exception of BJE1. The extracts purification with SPE method enhanced their influence on the GLUT5 protein expression by cells. This effect results from the efficient removal of non-phenolic compounds, such as organic acids, proteins, or unbounded sugars. More potent activity of the isolated phenolic compounds is in accordance with previously published data [37,41,42,43,44]. Still, our studies confirmed that *M. chamomilla* phenolic compounds potentially decreased the NBDF uptake by downregulation of GLUT5 level [22,33]. Villa-Rodriguez et al. (2017) showed that chamomile extract inhibited fructose transport across differentiated Caco-2 cells monolayers in a concentration-dependent manner primarily through GLUT2 inhibition, but with some effect on GLUT5. However, in literature, there are no reports on the effects of *A. graveolens* and *B. juncea* on fructose uptake and GLUT5 levels.

There are some reports distinguishing individual phenolic compounds as fructose uptake inhibitors [24,39,42], although there is no indication of their effect on the level of GLUT5 protein expression. For this reason, to distinguish phenolic compound/compounds potentially involved in GLUT5 level regulation and fructose uptake, we performed identification of phenolic constituents present in extracts purified with the SPE method (pAGE, pBJE1/2, pMCE). Analysis of the tested extracts is in agreement with other reports, showing that hydroxybenzoic and hydroxycinnamic acids, flavanols, flavonols and flavons were the dominant phenolic groups. pAGE was the richest source of flavanols with a high content of procyanidins C1 and B2, and the presence of EGCG, EGC, and (-)-epicatechin were confirmed in other studies [45,46]. Conversely to our results, in many of *A. graveolens* cultivars, Yao et al. (2010) identified additionally large amounts of apigenin, luteolin, and kaempferol [47]. In spite of this, we identified apigenin-7-*O*-glucoside as the main compound of *A. graveolens*, which is in agreement with Lin et al. (2007) [48]. Additionally, we indicated pAGE as a rich source of gallic acid, 3-coumaric acid, salicylic acid, ferulic acid, and 3,5-dicaffeoylquinic acid, which were identified also in other studies [45,46,49].

In agreement with other data, in purified extract of *M. chamomilla*, we identified chlorogenic acid, caffeic acid, 3-coumaric acid, salicylic acid, luteolin, apigenin, quercetin-3-*O*-glucoside [50,51,52], and ferulic acid [53]. However, the same reports show that chamomile is rich in apigenin-7-*O*-glucoside, rutin, quercetin, kaempferol, and isorhamnetin, which were not detected by us. On the other hand, in the purified pMCE extract we identified other compounds, such as quercetin-3-*O*-glucoside, isorhamnetin-3-*O*-glucoside, kaempferol-3-*O*-glucoside, and apigenin.

In the purified *B. juncea* extracts, apigenin was identified as the main phenolic compound. Other research indicated *B. juncea* as a source of apigenin [54,55], EGCG, rutin, caffeic acid, gallic acid, sinapic acid, ferulic acid, salicylic acid [32,56], kaempferol, quercetin, and isorhamnetin glycoside derivatives [57,58], which is in agreement with our results. Still, we have observed the difference between the profiles of phenolic compounds identified in both varieties of *B. juncea*. For example, in pBJE1, gallic and ferulic acids were detected as representatives of HBA and HCA compounds, whereas in pBJE2, other HBA and HCA compounds such as caffeic acid, salicylic acid, 3,5-dicaffeoylquinic acid, and sinapic acid were identified. Furthermore, kaempferol and isorhamnetin-3-*O*-glucoside were determined in pBJE1, while apigenin-7-*O*-glucoside and EGC were in pBJE2. Nevertheless, many publications indicate the type and conditions of breeding, seed origin, and ripening stage as factors influencing the qualitative and quantitative phenolic composition of plants [47,59,60,61].

It needs to be emphasized that this is the first investigation demonstrating a comparison of the effects of phenolic compounds from *A. graveolens*, *B. juncea*, and *M. chamomilla* on fructose uptake in Caco-2 cells with the involvement of GLUT5 transporter. Some reports showed that phenolic compounds or their mixture as a botanical preparation may have inhibitory activity on glucose and fructose influx into the enterocyte and circulation, and could partially contribute to the positive effects in metabolic disorders [62,63]. For example, grape juice rich in gallic acid, caffeic acid, quercetin-3-*O*-glucoside, quercetin-3-O-glucuronide, quercetin aglycone, and a number of anthocyanins reduced intestinal glucose and fructose transport by Caco-2 cell monolayers [64]. Among phenolics with proved inhibitory activity on glucose and fructose transporters is apigenin [24,65]-abundant phenolic compound, which was identified in pMCE and pBJE1/2. Kwon et al. (2007) showed that apigenin at a 50-µM concentration inhibited fructose uptake in Caco-2 cells [35]. The same research showed comparable activity for luteolin, which was identified in pMCE, and for quercetin, glycosides were identified in pMCE, pBJE1, and pBJE2. Inhibitory activity against fructose uptake in Caco-2 cells was observed by Lee et al. (2015) for quercetin (29.64 µg/mL), which modified GLUT5 transporter activity [21]. Other research published by Gauer et al., (2018) showed that apigenin (40 µM) and EGCG (72 µM) inhibited uptake of fructose into oocytes expressing GLUT5 protein, whereas quercetin had no significant effect [9]. While apigenin was identified as the GLUT5 inhibitor, kaempferol, rutin, caffeic acid, gallic acid, ferulic acid, chlorogenic acid, and EGCG were shown to inhibit fructose uptake by the GLUT2 transporter [66,67]. Andrade et al. (2017) suggested that above-mentioned phenolics interfered with fructose uptake (10–20% reduction). However, he has correlated this effect not only with GLUT5 but also with other GLUT transporter distinct from GLUT5 [23]. Taken together, the results presented and other reports suggest that inhibitory activity of fructose uptake in Caco-2 cells, which is followed by the reduction in GLUT5 protein and mRNA level, may be correlated with high phenolic content identified in the tested plant extracts, especially apigenin, quercetin glycosides, kaempferol, kaempferol-3-*O*-glucoside, EGCG, and ferulic acid. It can be speculated that the pAGE lack of influence on NBDF uptake, as well as on the level of GLUT5 protein, might be caused by the absence of apigenin and flavonols, which revealed inhibitory activity against fructose uptake. In order to confirm this hypothesis, we tested the relationship between the NBDF uptake with the main phenolic compounds content with Pearson’s correlation coefficients. The enhanced NBDF uptake was very strongly correlated with the concentration of procyanidin C1 (r = 0.982) and apigenin-7-*O*-glucoside (r = 0.881). Correlation analysis also evidenced a positive relationship between these compounds content and GLUT5 protein level with r = 0.870 for procyanidin C1, and r = 0.676 for apigenin-7-*O*-glucoside. Additionally, association of apigenin content with NBDF uptake with r = −0.462 demonstrates a moderate and negative correlation, where the increase in this compound concentration decreases the NBDF cellular uptake. In regard to the influence on GLUT5 protein expression, a comparable relationship was determined for kaempferol-3-*O*-glucoside and epigallocatechin-3-gallate presence with Pearson’s correlation = −0.729 and −0.796, respectively. Ferulic acid was identified as a strong negative regulator of expression of GLUT5 protein and uptake of fluorescent fructose analogue (r = −0.517 and r = −0.817, respectively). On the other hand, the Pearson’s coefficients r ≤ 0.184 suggest a weak correlation between the kaempferol content and both NBDF uptake and GLUT5 protein level. Despite demonstrated statistical results, it needs to be emphasized that studied extracts contained complex mixture of compounds which can also interact with each other. Thus, the presented correlations do not fully reflect the reactions and interactions between the compounds taking place in the studied mixture, and observed biological response can be regulated with involvement of different signaling pathways.

It is worth mentioning that, among factors influencing fructose uptake besides the regulation of the expression of GLUT5 protein, the transporter trafficking to the apical localization of intestinal cells is also important [10]. Thioredoxin-interacting protein (TXNIP) can physically bind to GLUT2 and GLUT5 in intestinal epithelial cells, and, in this latter case, it facilitates GLUT5 trafficking. It is known that TXNIP-elevated expression increases fructose uptake [68]. Moreover, recent data obtained in the mouse model revealed that deletion of Txnip in the intestinal epithelium reduced expression of *Slc2a5* gene-encoding GLUT5 protein [68]. Additionally, GLUT5 trafficking to the apical membrane is mediated by Ras-related protein-in-brain 11a (Rab11a)-dependent endosomes [51]. Txnip, by forming a complex with GLUT5 and Rab11a, may promote the transporter trafficking to the apical membrane and enhance fructose uptake [52]. Furthermore, TXNIP has been described as a possible link between cellular redox state and metabolism [69] since it plays a pivotal role in the process of ROS production, inducing oxidative stress and inflammation. Mice treatment with resveratrol reduced ROS generation, which was accompanied by suppression of Txnip and an increase in superoxide dismutase (SOD) activity [69]. In our study, Caco-2 cells treatment with extracts decreased intracellular ROS level. Based on these results, it can be supposed that phenolic components via antioxidant-based potential could inhibit the TXNIP activation by ROS as another pathway downregulating GLUT5 level.

It is worth underlining that GLUT5 expression is also regulated by transcription factor known as carbohydrate-responsive element–binding protein (ChREBP) [5]. This factor was upregulated in the mouse intestine during chronic consumption of high-fructose diet and elevated expression of GLUT5 [70]. There is a study suggesting that Txnip promotes the nuclear translocation of ChREBP factor, enhancing its binding to the promoter region of *Slc2a5* gene due to the presence of two carbohydrate response elements (ChoRE) for binding of ChREBP [71], as well as influences methylation of the *Slc2a5* gene [68]. ChREBP via ChoRE also regulates the expression of other genes encoding enzymes involved in fructose metabolism, such as ketohexokinase (KHK), which intensifies fructose metabolism-increasing hepatic lipogenesis [72]. Our results have indicated that, after Caco-2 cells treatment with pBJE1/2 and pMCE, the TXNIP and ChREBP mRNA levels were downregulated. Basing on these outcomes we can suspect that the reduction in the GLUT5, TXNIP, and ChREBP expression can be implicated in the mechanism responsible for decreased NBDF uptake by Caco-2 cells. To the best of our knowledge, this is the first study matching phenolic-rich extracts with their influence on fructose uptake with the involvement of TXNIP. However, one can presume that the GLUT5 expression is regulated by concerted action of several transcription factors which are, so far, only partially identified [14,73]. Recently, sterol-responsive liver X receptor α (LXRα) has been identified as a strong and specific regulator of human *GLUT5* promoter in DR4 site, along with thyroid hormone receptor (THR), which, probably together with ChREBP, is involved in fructose-stimulated *Slc2a5* transcription [14,73]. One cannot exclude an involvement of other transcription factors in regulation of the S*lc2a5* gene expression as it has been reported for some genes encoding enzymes involved in lipogenesis [74]. Moreover, previous studies indicate that GLUT5 expression is regulated not only at the transcriptional level, but also by diet-related signals activating epigenetic mechanisms [75]. It has been proven that GLUT5 up-regulation can be induced by fructose feeding, where fructose regulation increases polymerase II binding and histone H3 acetylation to the GLUT5 promoter, and induces acetylation of H3 and H4 proteins in *Slc2a5* gene and other fructose-inducible genes [74]. *B. juncea* and *M. chamomille* extracts are possible sources of constituents possessing the ability to alter histone acetyltransferase (HAT), histone deacetylase (HDAC), or histone methyltransferase (HMT) activities [76,77]. Numerous studies indicate apigenin as the factor which modulates activity of histone modifiers. It was demonstrated that, in prostate cancer cells, apigenin inhibited growth by class I histone deacetylase alteration [78], and induced the demethylation of nuclear factor erythroid 2-related factor 2 (Nrf2) promoter, leading to the upregulation of Nrf2 mRNA [79]. Nrf2 factor modulates activity of genes responsible for regulation of red-ox processes, metabolism of xenobiotics, DNA repair, lipid and carbohydrate metabolism, and the iron homeostasis. Moreover, apigenin via interaction with Kelch-like ECH-associated protein 1 (Keap1) simultaneously blocked Keap1 binding with Nrf2, which increased expression of genes encoding proteins, preventing metabolic alterations induced by fructose [80]. It can be suspected that GLUT5 expression can be modulated by phenolic compounds acting at the levels of epigenetic modifications; however, this mechanism requires more detailed studies.

On the other hand, amongst GLUT transporters, only GLUT 5 is specific for fructose and exhibits no ability to transport other carbohydrates [81]. Despite significant progress in the studies on the structure and function of GLUT5 [8], still little is known about the determinants of substrate and inhibitor specificity of this protein. In particular, the mechanism of inhibition of GLUT5-mediated fructose transport is not yet fully understood. Molecular docking simulation techniques offer a fast, cheap, and easy way to predict the binding energies and geometries of the binding sites in the receptor molecule. In particular, these theoretical methods allow to assess the affinity of various ligands, including potential substrates and inhibitors, to the receptor, as well to indicate the residues crucial for ligand recognition. However, it should be noted that the theoretically predicted binding affinities should be interpreted with great caution. Due to the roughly approximations in the applied model, which is practically restricted only to the individual receptor-ligand system, and negligence of environmental effects (such as solvent effects for example), it is hard to expect that the obtained binding energies will be in excellent agreement with experimental findings. The extract is the mixture of various compounds of different, but comparable inhibitory potential against GLUT5, therefore competitive inhibition mechanism is expected in the real systems. Moreover, intermolecular interactions between individual components of extract should also be expected, and these are not taken into account in the applied theoretical model. In other words, the observed inhibitory effect of the extract on fructose uptake is the resultant of: (1) competitive interactions of many compounds of a different binding affinity to GLUT5 transporter (competitive inhibition); (2) the intermolecular interactions between various individual ligand–ligand pairs, despite of the rough approximation in the applied semi-empirical model in the determination of binding affinities and binding site geometries of the tested ligands in GLUT5, as well as the negligence of solvent/environmental effects [82,83]; and (3) restriction of the theoretical predictions to individual 1:1 protein–ligand complex, our results of molecular docking simulations provided additional unique information, which may contribute to understanding molecular basis of substrate and inhibitor specificity of GLUT5.

Molecular docking simulations of fluorescent fructose analogue NBDF (used in fructose uptake experiments), selected compounds identified in polyphenolic extracts: kaempferol; kaempferol-3-*O*-glycoside; apigenin; apigenin-7-*O*-glucoside; epigallocatechin-3-gallate (ECGC); and procyanidin C1, as well as known GLUT5 inhibitor MSNBA to bovine GLUT 5 (which exhibits ~81% sequence identity to human GLUT5 [8] located all the ligands tested bound close to the fructose binding site, since most of the interacting residues were those lining the central fructose binding site of GLUT5 (Ile170, Ile174, Gln167, Gln288, Gln289, Asn325, Trp419, Tyr32, His387, Ser392 [8]. Interestingly, both the fluorescent substrate analogue NBDF, as well as all the tested potential inhibitors were found to interact with Gln167 and most of them (with the exception of procyanidin C1 and ECGC) also did so with Ile170 (Table 3). These residues, according to the previous study [8,20], are considered as the key residues for fructose transport. For rat GLUT5, the replacement of Gln166, an equivalent of Gln167 in bovine GLUT5, has been reported to alter the substrate binding specificity from fructose to glucose GLUT5-mediated transport [8]. Apparently, subtle sequence differences are responsible for ligand specificity. On the basis of molecular dynamics simulations previously performed by Ebert K. et al. with the use of chimeric proteins of GLUT5 and GLUT7 [20], the authors hypothesized that Gln167E mutation triggered the intracellular loop to act as a “gate” or “lid” that blocks fructose transport [20].

Based on the theoretically predicted binding energies, which were in the range of 10 kcal/mol, it may be expected that the substrate and potential inhibitors may compete with each other for a binding site at the GLUT5 active site. However, our results showed higher binding affinity of the potential inhibitors to GLUT5 as compared to that for the fluorescent substrate analogue. In particular, amongst the tested ligands, procyanidin C1 was predicted to show the highest affinity to GLUT5 (that is the more negative value of binding affinity).

It should be stressed that, although the lowest value of binding affinity indicates the greatest stability of the resulting complex with the strongest intermolecular interactions, it does not unequivocally mean that these interactions will eventually result in the inhibitory effect. This is because the peculiar interactions involving unique receptor residues and inhibitor are crucial determinants for the resulting inhibitory effect. Therefore, strong intermolecular interactions in the GLUT5-procyanidin C1 complex, indicated by the lowest value of binding energy, does not necessary lead to a strong inhibitory effect. The observed low inhibitory effect of extract rich in procyanidin C1 on fructose uptake may, at least partly, be explained by the specific geometry of the GLUT5-procyanidin C1 intermolecular complex obtained from molecular docking simulations. Inspection of the geometries of GLUT5-procyanidin C1 complex revealed no hydrogen bonding interactions. Additionally, no Ile residue, which was previously indicated as one of the determinants of GLUT5 specificity to fructose [20], was found amongst the interacting residues in the theoretically predicted procyanidinC1 binding site. It may be speculated that large molecule sizes of procyanidin C1 make this ligand act as steric hindrance impeding the access of other inhibitors of higher inhibitory potential to the binding site in GLUT5. It is also tempting to suggest that binding of this ligand to GLUT5 does not eventually lead to the inhibition of GLUT5.

Taken together, presented results suggest that *Brassica juncea* phenolic compounds, as well as *Matricaria chamomilla*, were able to decrease the fluorescent fructose analogue uptake in Caco-2 cells and could be potentially used in further research dedicated to phytocompounds able to protect against the development of metabolic disorders resulting from excessive fructose intake.

## 4. Materials and Methods

### 4.1. Preparation of Plant Extracts

The seeds of root celery *Apium graveolens* L. (var. rapaceum, Talar) were bought from W. Legutko Sp. z o.o., Jutrosin, Poland, while *Brassica juncea* (var. Green giant) and *Brassica juncea* (var. Red giant) were bought from P.W. Cleome, Czestochowa, Poland, and the plants were grown under greenhouse conditions. Dried flowers of *Matricaria chamomilla* were bought from Dary Podlasia, Bielsk Podlaski, Poland. Plant material was cut into small pieces, which were then grinded, freeze-dried, and extracted with a 70% (*v*/*v*) ethanol solution on the stirrer at 800 rpm for 30 min. To remove organic solvents, the supernatants were concentrated at 40 °C in the vacuum rotary evaporator (RII, Büchi, Switzerland), and then water concentrates were freeze-dried to afford ethanol extracts. In order to obtain purified extracts rich in phenolic compounds, the purification process was carried by solid-phase extraction with C-18 Sep-Pak cartridge (10 g capacity, Waters Corp., Milford, MA, USA), which were then pre-treated with the methanol and water mixture. After being dissolved in water, freeze-dried extracts were passed through the column, while phenolic compounds were bound to the C-18 cartridge and sugars, and other polar compounds were removed with water. In the next step, phenolic compounds were eluted with methanol and, after removing the organic solvent under vacuum, the dry residue was diluted in water and freeze-dried to afford purified extracts (pAGE, pMCE, pBJE1, pBJE2). For biological activity assays, extracts were dissolved in a PBS/dimethyl sulfoxide (DMSO) (1:1 *v*/*v*) at concentration 100 mg/mL.

### 4.2. Determination of Phenolic Compounds Composition by UPLC Method

Phenolic profiles were determined using an ACQUITY Ultra Performance LC system (UPLC) equipped with a photodiode array detector with a binary solvent manager (Waters, Milford, MA, USA). Separation was achieved on a Acquity on a Acquity UPLC HSS T3 column (150 × 2.1 mm, 1.8 µm; Waters). The mobile phase was a binary gradient with A, water/formic acid (95:5:4.5, *v*/*v*), and B, acetonitrile, with a flow rate of 0.45 mL/min [84]. The binary gradient was as follows: initial conditions—99% A (0 min), 12 min—75% A, 12.5 min—100% B, 13.5 min—99% A (12.5–13.5 min). The runs were monitored at the following wavelengths: flavanols at 280 nm, hydroxycinnamic acids at 320 nm, flavonols at 360 nm, and anthocyanins at 520 nm. The retention times and spectra were compared to those of the authentic standards. Calibration curves at concentrations ranging from 0.06 to 2 mg/mL (r2 ≥ 0.96) were made from gallic acid, salicylic acid, chlorogenic acid, caffeic acid, 3-coumaric acid 3,5-dicaffeoylquinic acid, ferulic acid, sinapic acid, procyanidin B2, procyanidin C1, (-)-epicatechin, epigallocatechin-3-gallate, epicatechin-3-gallate, rutin, quercetin-3-*O*-glucoside, quercetin-3-Oglucuronide, kaempferol, kaempferol-3-*O*-glucoside, isorhamnetin-3-*O*-glucoside, isorhamnetin-3-*O*-rutinoside, apigenin, apigenin-7-*O*-glucoside, and luteolin. The data was collected by Mass-Lynx™ V 4.1 software. The results are expressed as mg of phenolic compounds per g of freeze-dried extract.

The purified VOJ were analyzed for the composition of phenolic compounds by the UPLC method, as described previously [37,44]. Quantitatively, the main component of the preparations was chlorogenic acid, which accounted for 78.05% of total content of identified phenolic compounds in purified VOJ. Additionally, in VOJ, other hydroxycinnamic acid derivatives were identified (caffeoylquinic acid and its derivatives, feruoylquinic acid derivatives), as well as flavanols such as procyanidin B1, procyanidin B2, (+)-catechin, (-)-epicatechin, gallocatechin gallate, procyanidin C1, quercetin glycosides (flavonols), and anthocyanins-cyanidin glycosides.

### 4.3. Cell Culture and Exposure Conditions

Human colon adenocarcinoma cell line Caco-2 cell line was obtained from ATCC, (Manassas, VA, USA). Cells were grown in DMEM with a 10% fetal bovine serum (FBS) medium supplemented with 100 U/mL penicillin, 100 µg/mL streptomycin, and 25 µg/mL amphotericin B. All cell culture experiments were performed in a humidified 5% CO_2_ and 95% atmosphere at 37 °C. Cells were in cubated with tested extracts for 24 h. Tested lyophilizates were dissolved in a phosphate-buffered saline (PBS)/dimethylsulfoxide (DMSO) (1:1 *v*/*v*) at concentration 100 mg/mL and were further diluted with culture medium. The extract’s concentrations used in biological studies are presented in the descriptions of the tests carried out.

All the experimental measurements were performed using the Synergy 2 BioTek Microplate Reader (BioTek, Winooski, VT, USA). All cell culture reagents were obtained from Life Technologies (Carlsbad, CA, USA).

### 4.4. Cell Viability

Metabolic activity was evaluated with fluorescent measurements with PrestoBlue (Life Technologies, Carlsbad, CA, USA), according to the manufacturer’s instructions. Cells were seeded into 96-well plates at 1 × 10^4^ cells/well density in complete medium and grown overnight and then incubated in the presence of studied samples for another 24 h. After this, fluorescent reagent was added for 30 min, and the fluorescent signal at F530/590 nm was measured.

### 4.5. Detection of Intracellular Reactive Oxygen Species Generation

The effect of samples on intracellular generation of reactive oxygen species (ROS) was investigated using dichloro-dihydro-fluorescein diacetate (DCFH-DA) chemical. Cells were seeded into a 96-well plate at a density of 1 × 10^4^ cells/well overnight. After 24 h, tested preparations were added, and cells were incubated with samples for another 24 h. After the cells’ treatment with preparations, the cells were washed with phosphate-buffered saline (PBS) and incubated with 10 µM DCFH-DA for 30 min. For positive control, *tert*-BOOH (*t*-BOOH) was used at a concentration of 500 µM. Fluorescent signal at F485/530 nm was analyzed.

### 4.6. Western Blot Analysis

The experiment cells were seeded into a 6-well plate at density of 2 × 10^6^ cells/well. After 24 h, tested compounds were added for 36 h (cytotoxic effect was not observed). To prepare the total lysates, monolayers of Caco-2 cells were scraped and lysed in Mammalian Protein Extraction Reagent (M-PER) containing a protease and phosphatase inhibitors cocktail (Thermo Scientific, Waltham, MA, USA). Cell lysates were separated after centrifugation at 13,000 rpm for 5 min at 4 °C. The total protein quantification was measured using the Protein Assay Dye Reagent Concentrate (Bio-Rad Laboratories BmbH, München, Germany). Each 30 µg of protein were separated by 15% sodium dodecyl sulphate-polyacrylamide gel electrophoresis (SDS-PAGE). Proteins were transferred to 0.45 µm nitrocellulose blotting membrane (GE Healthcare, Chicago, IL, USA) using Tranfer-Blot®-Turbo™ transfer system (Bio-Rad Laboratory, Hercules, CA, USA). The membranes were blocked using 5% bovine serum albumin (BSA) in phosphate-buffered saline containing 0.1% Tween-20 (PBST) for 2 h at room temperature and then incubated with primary antibodies overnight at 4 °C. Primary rabbit antibodies targeting GLUT5 were purchased from Invitrogen (Carlsbad, CA, USA) and diluted at a ratio of 1:1000 in PBST and GAPDH from Sigma-Aldrich and diluted at a ratio of 1:2000 in PBST (Seinheim, Germany). Afterwards, the membranes were washed five times with PBST and incubated with horseradish peroxidase (HRP)-conjugated secondary anti-rabbit antibodies (Cell Signaling, Danvers, MA, USA) diluted at a ratio of 1:3000 in 5% nonfat dry milk in PBST for 1 h at room temperature. After that, membranes were washed five times with PBST. The proteins were visualized using an enhanced chemiluminescent SuperSignal West Pico Trial Kit (Thermo Scientific, Waltham, MA, USA). For acquisition and densitometric analysis of Western blot images, the ChemiDoc™MP Image System with Image™ 5.1 software (Bio-Rad Laboratories, Hercules, CA, USA) was used. Relative protein bands intensity was normalized to GAPDH and quantified with respect to control (untreated) cells.

### 4.7. DNA Damage and Repair

The research was conducted according to Zakłos-Szyda et al. (2020). Firstly, Caco-2 cells were damaged with 25 µM H_2_O_2_ and 6.8 μM MNNG for 10 min on ice and then DNA repair was investigated under the exposition (for 60 and 120 min, 37 °C) to the 0.25 mg/mL concentration of extracts (evaluated earlier as non-cyto- and genotoxic). At initial and after 60 and 120 min incubation, aliquots of the suspensions were taken and the samples were placed in an ice bath (to stop DNA repair). At each time interval, an alkaline comet assay (pH > 13) was performed and DNA repair was quantified by determination of the extent of residual DNA damage. Two control samples were included: two positive-cells exposed to H_2_O_2_ or MNNG, and one negative-cells in DMEM. The concentration of cells in each sample was adjusted to 105/mL. After each interval, aliquots of cells were centrifuged (182× *g*, 15 min, 4 °C), decanted, suspended in LMP agarose (0.75%), and distributed onto slides precoated with NMP agarose (0.5%) and immersed in lysing buffer (2.5 M NaCl, 100 mM EDTA, 10 mM Tris, 1% Triton X-100). Next, DNA was allowed to unwind (20 min) in the buffer (300 mM NaOH, 1 mM EDTA) and slides were subjected to horizontal gel electrophoresis in an electrophoretic buffer (30 mM NaOH, 1 mM EDTA). Electrophoresis was conducted at 4 °C for 30 min. Slides were stained with 1 mg/mL DAPI. Comets were visualized at 200× magnification in a fluorescence microscope (Nikon Eclipse Ci H600L, Japan) connected to personal computer-based image analysis system Lucia-Comet v. 7.0 (Laboratory Imaging, Prague, Czech Republic). Fifty images were randomly selected from each sample and the percentage of DNA in the comet tail was measured. The results were presented as mean ± standard error of the mean (S.E.M.). Two-way analysis of variance (ANOVA) was conducted using OriginPro 6.1 software to evaluate the experimental data. Significant differences were accepted at *p* < 0.05, as calculated with Duncan’s multiple range test (Statistica 10, StatSoft).

4′,6-diamidino-2-phenylindole (DAPI), EDTA, H_2_O_2_, low melting point (LMP) agarose, methylnitronitrosoguanidine (MNNG), NaCl, NaOH, normal melting point (NMP) agarose, Tris, and Triton X-100 were purchased from Sigma-Aldrich (St. Louis, MO, USA).

### 4.8. Gene Expression Analysis

To study the influence of extracts on gene expression, Caco-2 cells were seeded into a 6-well plate at a density of 2 × 10^6^ cells/well and grown overnight. After 24 h, tested compounds were added for another 24 h. Total RNA was extracted from cells using GeneMatrix Universal RNA Purification Kit (Eurex Ltd., Gdansk, Poland), according to the manufacturer’s procedure. RNA samples were purified with Amplification Grade DNase I and reverse transcribed with NGdART RT Kit (Eurex Ltd., Gdansk, Poland). Real time RT-PCR was carried out using SG qPCR Master Mix (Eurex Ltd., Gdansk, Poland) on a BioRad CFX96 qPCR System (Bio-Rad, Hercules, CA, USA). Complementary DNA, representing 6 ng total RNA per sample, was subjected to 25–40 cycles of PCR amplification. Samples were first incubated at 95 °C for 40 s, then at 55 °C for 30 s, and finally at 72 °C for 30 s. To exclude non-specific products and primer-dimers, after the cycling protocol, a melting curve analysis was performed by maintaining the temperature at 55 °C for 2 s, followed by a gradual temperature increase to 95 °C. The threshold cycle (Ct) values for that gene did not change in independently performed experiments. The level of target gene expression level was calculated as 2^-ΔΔCt^, where ΔΔCt = [Ct(target) − Ct(GAPDH)]_sample_ − [Ct(target) − Ct(GAPDH]_calibrator_. Gene expression was normalized using constitutively expressed glyceraldehyde-3-phosphate dehydrogenase (GAPDH) as a reference gene. The following primer sequences were used to determine the genes’ expression: GAPDH: 5′-CCACCCATGCAAATTCCATGGCA-3′ (F) and 5′-TCTAGACGGCAGGTCAGGTCCACC-3′ (R); GLUT5: 5′-ACCGTGTCCA- TGTTTCCATT-3′ (F) and 5′-ATTAAGATCGCAGGCACGAT-3′ (R); TXNIP: 5′-CTTAGTGTAACCAGCGGCGT-3′ (F) and 5′-CTGAGGAAGCTCAAAGCCGA-3′ (R); ChREBP: 5′-CCAGACAGCAACAAGACCGA-3′ (F) and 5′-CTGGTCAAAA- CGCTGGTGTG-3′ (R).

### 4.9. NBDF Uptake

Cells were seeded into 96-well plate at a density of 1 × 10^4^ cells/well and incubated for 24 h. Briefly, after 24 h of treatment samples, 100 µM of fluorescent fructose analogue 1-(7-nitro-1,2,3-benzadiazole)-fructose (NBDF, Cayman Chemical, Michigan, USA) was added in glucose- and serum-free medium. After 3 h of incubation with NBDF, cells were washed twice with serum- and glucose-free medium, and fluorescent signal at F485/530 nm was measured immediately. As a GLUT5 inhibitor, 50 µM MSNBA was used (CymitQuimca, S.L., Barcelona, Spain).

### 4.10. Molecular Docking Simulations

Molecular docking was performed with the use of Autodock Vina software [85,86]. The pdb input file of the crystal structure (open inward-facing conformation) of bovine fructose transporter GLUT5 (4YB9) was retrieved from the Protein Data Bank database [https://www.rcsb.org/] accessed on 3 August 2021. The input structure for the receptor (GLUT5) was prepared for molecular docking using Chimera software [87] by removing co-crystallized ligands, crystal waters, and other heteroatoms and by adding Gasteiger charges and polar hydrogens.

The input structures for the ligands: procyanidin C1, apigenin, apigenin-7-*O*-glucoside, kaempferol, kaempferol-3-*O*-glycoside, epigallocatechin-3-gallate (ECGC) were downloaded from PubChem database [https://pubchem.ncbi.nlm.nih.gov/] accessed on 3 August 2021. The obtained files were converted to pdb file using the OpenBabel tools [88].

The structures of *N*-[4-(methylsulfonyl)-2-nitrophenyl]-1,3-benzodioxol-5-amine (MSNBA) and 1-deoxy-1-[(7-nitro-2,1,3-benzoxadiazol-4-yl)amino]-d-fructose (NBDF) were optimized with the use of Gaussian 09 and GaussView 5.0 [89] software applying the PM6 semi-empirical method. The analysis of the PM6 that calculated vibrational frequencies was performed, and no imaginary vibrational frequency was found which indicated that the optimized geometries corresponded to the local minima on the potential energy hypersurface.

The first step of docking simulations was performed with searching space (grid box) which encompassed the whole structure of the receptor. Subsequently, the search space was restricted only in the targeted binding sites [8]. The resulting docking solutions were subsequently clustered with a root-mean-square deviation (rmsd) tolerance of 2.0 Å and were ranked by binding energy values. The lowest binding energy conformer was searched out of ten different conformers for each docking simulation. AutoDock 4.2 software was used to visualize the docking conformations and the binding sites.

### 4.11. Statistical Analysis

Unless stated otherwise, all the biological results are presented as means of 3–6 repeated experiments ± SEM. All calculations were evaluated for significance using one-way ANOVA followed by Dunnett’s test with the GraphPad Prism 6.0 software (GraphPad Software, Inc., La Jolla, CA, USA). *p* ≤ 0.05 was considered statistically significant. Pearson’s correlation coefficients were determined using Microsoft Excel XP.

## 5. Conclusions

In this study, we examined the effect of four phenolic-rich extracts obtained from *A. graveolens*, *B. juncea*, and *M. chamomilla* on fluorescent fructose analogue (NBDF) uptake by Caco-2 cells. Whereas *A. graveolens* effectively decreased intracellular ROS production, it had no effect on fructose uptake. As the most effective reducers of fructose uptake, the extracts purified with the SPE method from *B. juncea* and *M. chamomilla* were identified. The chemical characterization showed that these reducers of NBDF uptake contain large amounts of apigenin, flavonols (i.e., kaempferol-3-*O*-glucoside), and a low quantity of procyanidin C1 and apigenin-7-*O*-glucoside. Moreover, we have observed that these extracts downregulated the levels of GLUT5 protein and mRNAs encoding ChREBP and TXNIP transcription factors. Therefore, one can hypothesize an involvement of these two transcription factors in the regulation of the *Slc2a5* gene expression. The results of theoretical predictions applying molecular docking simulation have suggested that hydrogen bonding interactions incorporating kaempferol, kaempferol-3-*O*-glycoside, apigenin, and apigenin-7-*O*-glucoside play an important role in inhibition of GLUT5-mediated fructose transport. The molecular docking simulation has located the tested ligands inside the fructose active center, and indicated that, among the amino acid residues in GLUT5 interacting with the ligands, Gln167 and Ile170 are likely to be the key residues critical for recognition of inhibitor. It can be suspected that some of *B. juncea* and *M. chamomilla* constituents, such as apigenin, are able to alter GLUT5 expression via epigenetic regulation; however, this statement requires a more detailed study. Despite this, the results obtained contribute to a better understanding of GLUT5 function and its regulation by phytocompounds present in the human diet. Still, future studies should be focused on the extracts activity after their in vitro digestion process. Taken together, presented results suggest that *Brassica juncea* phenolic compounds, as well as *Matricaria chamomilla*, were able to decrease the fluorescent fructose analogue uptake in Caco-2 cells and could be potentially used in further research dedicated to phytocompounds able to protect against the development of metabolic disorders resulting from excessive fructose intake.

## Figures and Tables

**Table 1:** The IC_0_ concentration values for the extracts determined with PrestoBlue assay after 24-h incubation of Caco-2 cells.

Extract	AGE	pAGE	BJE1	pBJE1	BJE2	pBJ2	MCE	pMCE
IC_0_ [mg/mL]	>2.50	0.75	2.00	0.25	2.50	0.75	>2.50	0.25

AGE-*Apium graveolens* extract, BJE1/2-*Brassica juncea* extract 1/2, MCE-*Matricaria chamomilla* extract; p-the extract purified with solid-phase extraction.

**Table 2:** Individual phenolic compounds content in the extracts from *Apium graveolens* (pAGE), *Matricaria chamomilla* (pMCE), and *Brassica juncea* 1/2 (pBJE1/2) purified by solid-phase extraction.

The Compound Identified	λ_max_ [nm]	Rt	Quantification [mg/g of Extract]			
			pAGE	pMCE	pBJE2	pBJE1
HYDROXYBENZOIC AND HYDROXYCINNAMIC ACIDS						
Gallic acid	270	2.0	12.68 ± 0.00 *^b^*	n.d.	n.d.	3.96 ± 0.10 *^a^*
Chlorogenic acid	324	4.88	n.d.	0.96 ± 0.01	n.d	n.d.
Caffeic acid	324	6.50	n.d.	0.81 ± 0.00 *^a^*	2.02 ± 0.04 *^b^*	n.d.
3-Coumaric acid	277	9.24	6.24 ± 0.30 *^b^*	0.17 ± 0.01 *^a^*	n.d.	n.d.
Salicylic acid	303	10.70	41.88 ± 3.19 *^c^*	28.00 ± 0.18 *^b^*	24.63 ± 2.17 *^a^*	n.d.
3,5-Dicaffeoylquinic acid	326	10.85	6.23 ± 0.02 *^a^*	n.d.	12.85 ± 0.31 *^b^*	n.d.
Ferulic acid	315	11.90	5.19 ± 0.01 *^a^*	36.20 ± 0.08 *^c^*	17.78 ± 1.41 *^d^*	7.25 ± 0.04 *^b^*
Sinapic acid	326	12.20	n.d.	n.d.	6.40 ± 0.22	n.d.
FLAVANOLS						
Procyanidin B2	286	6.51	7.11 ± 0.67	n.d.	n.d.	n.d.
(-)-Epicatechin	284	7.00	6.27 ± 0.04	n.d.	n.d.	n.d.
Procyanidin C1	278	7.87	256.76 ± 6.74 *^c^*	n.d.	2.32 ± 0.10 *^a^*	70.43 ± 9.94 *^b^*
Epigallocatechin-3-gallate	274	9.50	6.30 ± 0.01 *^b^*	4.94 ± 0.17 *^a^*	51.62 ± 0.18 *^c^*	n.d.
Epicatechin-3-gallate	276	11.23	6.51 ± 0.24	n.d.	n.d.	n.d.
FLAVONOLS						
Rutin	355	9.70	n.d.	n.d.	25.28 ± 1.53 *^b^*	4.98 ± 0.02 *^a^*
Quercetin-3-*O*-glucoside	350	9.91	n.d.	1.90 ± 0.03 *^b^*	27.70 ± 0.47 *^c^*	0.45 ± 0.13 *^a^*
Quercetin-3-*O*-glucuronide	350	9.98	n.d.	n.d.	22.20 ± 0.64	n.d.
Kaempferol-3-*O*-glucoside	353	10.75	n.d.	6.21 ± 0.04 *^b^*	18.56 ± 0.53 *^c^*	10.40 ± 0.08 *^a^*
Isorhamnetin-3-*O*-rutinoside	345	12.20	n.d.	n.d.	n.d.	4.72 ± 0.22
Isorhamnetin-3-*O*-glucoside	352	12.42	n.d.	20.17 ± 0.44 *^c^*	1.05 ± 0.12 *^a^*	9.83 ± 0.26 *^b^*
Kaempferol	352	12.98	n.d.	n.d.	n.d.	30.97 ± 0.14
FLAVONS						
Apigenin-7-*O*-glucoside	336	13.00	302.01 ± 14.43 *^b^*	n.d.	43.66 ± 1.65 *^a^*	n.d.
Luteolin	369	13.18	n.d.	6.45 ± 0.46	n.d.	n.d.
Apigenin	336	13.20	n.d.	40.47 ± 1.01 *^a^*	199.12 ± 0.85 *^c^*	165.81 ± 0.45 *^b^*
TOTAL PHENOLICS	-		680.20 ± 22.42 *^d^*	153.07 ± 0.71 *^a^*	455.19 ± 2.61 ^c^	308.79 ± 9.79 *^b^*

n.d.-not detected; results are expressed as a mean ± standard deviation (*n* = 3); the values expressed with the different superscript letter (*a*, *b*, *c*) within the same raw differ significantly (one-way ANOVA and Duncan’s test, *p* ≤ 0.05).

**Table 3:** Values of binding affinities and residues of GLUT5 in close contact with ligand: NBDF, MSNBA, and selected phenolic compounds identified in extracts.

Ligand	Binding Affinity		Residues Interacting with a Ligand
	kcal/mol	kJ/mol	
Kaempferol	−7.7	−32.2	Gln288 *, Gln289, Ile170, Ser143, His419, Gln167 *
Kaempferol-3-*O*-glycoside	−9.4	−39.3	Pro147, Asn144, Ser143, Ser29, Ile170, Gln167 *, Trp420, His419, Tyr412, Gly416, Met285, Gln288 *, Ala396
Apigenin	−7.6	−31.8	Gln289 *, Ile170, Ser143, His419, Gln167, Tyr32, Asn294
Apigenin-7-*O*-glucoside	−9.3	−38.9	Ser392, Gln167, Tyr32, Ile170, Pro166, His419, Gln288 ^*^, Asn294 ^*^
Epigallocatechin-3-gallate (ECGC)	−8.6	−35.9	Ser143, Asn144, Tyr412, Gly416, Pro147, Trp420, Pro166, His419,Gln167, Gly163
MSNBA	−8.4	−35.1	Tyr297, Asn325 *, Val293, Asn294, Gln289 *, Gln288 *, Ala396, Gln167, Ile170, Ser392, His419, Tyr32
Procyanidin C1	−11.4	−47.7	Gly416, Tyr412, Arg408, Ala396, Ser143, Ser142, Ser29, Pro147, Pro166, Leu162, Pro393, Gln167, Met148, Thr400, Val146
NBDF	−7.5	−31.4	Gln167, His419 ^*^, Ile170, Tyr32, Trp420,Asn294,Gln289

* residues involved in hydrogen bonding interactions.

## Data Availability

Not applicable.

## Sample Availability

Samples of the compounds are not available from the authors.
